# *Bifidobacterium breve* MN15965 Improved Bacterial Diversity, Short-Chain Fatty Acid Production, and Immune Activation in a Cyclophosphamide-Induced Immunosuppression Mouse Model

**DOI:** 10.3390/microorganisms14050949

**Published:** 2026-04-23

**Authors:** Tinghao Liu, Xinyi Zhao, Yan Hui, Jing Yang, Jianqiang Li, Haisang Qin, Ke Zhao, Jinjun Li, Xiangyu Bian, Xin Wang, Yuling Li, Fangshu Shi, Yuejian Mao, Xiaoqiong Li

**Affiliations:** 1 State Key Laboratory for Quality and Safety of Agro-Products, Institute of Food Science, Zhejiang Academy of Agricultural Sciences, Hangzhou 310021, China; l2247644680@gmail.com (T.L.); 15757237055@163.com (X.Z.); jianqiang.li910@outlook.com (J.L.); qinhaisang@163.com (H.Q.); zhaoke@zaas.ac.cn (K.Z.); lijinjun@zaas.ac.cn (J.L.); bianxiangyu@zaas.ac.cn (X.B.); xxww101@sina.com (X.W.); liyuling817@163.com (Y.L.); shifangshu@zaas.ac.cn (F.S.); 2 School of Public Health and Nursing, Hangzhou Normal University, Hangzhou 311121, China; huiyan@hznu.edu.cn; 3 Global R&D Innovation Center, Inner Mongolia Mengniu Dairy (Group) Co., Ltd., Hohhot 011500, China; yangjing1119@mengniu.cn

**Keywords:** *Bifidobacterium breve*, cyclophosphamide, gut microbiota, immunology, short-chain fatty acids (SCFAs), probiotic

## Abstract

The gut microbiota serves as a critical interface for host immunity, making it a promising target for probiotic intervention. In this study, we investigated the immunomodulatory potential of the strain *Bifidobacterium breve* (*B. breve*) MN15965 and the underlying role of gut bacterial communities in this process. We first assessed its in vitro immunomodulatory activity by measuring nitric oxide and cytokine secretion in THP-1 macrophages. Subsequently, an immunosuppressed mouse model was established by treating BALB/c mice with cyclophosphamide (CTX), a chemotherapeutic agent known to cause immune dysfunction and mucosal damage. In this model, we performed a series of analyses, including H&E staining, measurement of hematological parameters and serum cytokines/immunoglobulins, quantification of fecal short-chain fatty acids (SCFAs) by gas chromatography, and profiling of gut microbiota composition via 16S rRNA gene amplicon sequencing. The results showed that MN15965 supernatant enhanced TNF-α, IL-1β, and GM-CSF secretion in THP-1 cells, promoting M1 macrophage activation in vitro. In the in vivo model, MN15965 administration restored spleen and thymus tissue integrity and improved physiological indices, hematological parameters, and immunoglobulin levels. Furthermore, MN15965 increased fecal SCFAs, particularly butyric and valeric acid, increased gut bacterial diversity, and enriched potentially beneficial SCFA-producing taxa, including *Lachnospiraceae* and *Eubacterium*. These findings demonstrate that *B. breve* MN15965 alleviated CTX-induced immunosuppression by activating immune responses, regulating gut bacterial communities, and boosting SCFA production.

## 1. Introduction

The gut microbiota is integral to host health, playing a pivotal role in the development and homeostasis of the immune system [1,2]. This regulation is largely mediated by short-chain fatty acids (SCFAs), i.e., acetic acid, propionic acid, butyric acid, and valeric acid [3,4]. Disrupted SCFA production accompanied by gut microbiota dysbiosis is reported to contribute to the pathogenesis of autoimmune diseases like inflammatory bowel disease, rheumatoid arthritis, celiac disease, and systemic lupus erythematosus [5]. Cyclophosphamide (CTX) is widely used in chemotherapy but is known to cause severe immunosuppression and intestinal mucositis [6]. CTX administration can exacerbate gut dysbiosis [7], damage the intestinal epithelial barrier by disrupting tight junction proteins [8], and increase intestinal permeability, leading to the translocation of pathogens [9]. These disruptions collectively result in reduced immune organ function, hematopoietic abnormalities, and disrupted gut microbial diversity. Understanding this cascade underscores the potential of microbiota-targeted strategies to restore immune homeostasis. Consequently, the CTX-induced immunosuppression model serves as a well-established and relevant system for evaluating the immune-activating effects of probiotic interventions [10].

Probiotic supplementation represents a promising strategy to counteract such dysbiosis and bolster host immunity. Accumulating evidence has highlighted the efficacy of various probiotic strains in alleviating chemotherapy-induced intestinal toxicity [4,11,12]. Among probiotic candidates, *Bifidobacterium* spp. are particularly noteworthy. As keystone commensals in the healthy infant gut, bifidobacteria facilitate the establishment of other butyrate-producing bacterium (e.g., *Anaerostipes caccae* L1–92) and indirectly modulate host physiology [13]. Critically, early-life colonization by *Bifidobacterium* is linked to a lower incidence of immune-mediated diseases, whereas its depletion in both clinical and experimental settings correlates with immune dysregulation [14]. Beyond their critical role in infants, *Bifidobacterium* spp. are considered to be a probiotic source due to their ability to maintain intestinal barrier integrity and modulate systemic immune homeostasis [15]. Notably, specific strains such as *Bifidobacterium breve* (*B. breve*) M-16V exhibit well-characterized immunomodulatory capacities, including allergy prevention and immune balance restoration [16]. Similarly, *B. longum* 1714 mitigates peripheral inflammation through multiple mechanisms [17].

The interplay between probiotic strains such as *Bifidobacterium*, the gut microbiome, and host immunity constitutes a dynamic, mutually influential network [18]. As keystone taxa, bifidobacteria contribute to immune modulation in part by promoting the production of microbial metabolites, including SCFAs and tryptophan derivatives [19]. They shape the gut ecosystem by outcompeting pathogens and facilitating the growth of other commensal SCFA producers through mechanisms like cross-feeding [13]. These resulting microbial metabolites, particularly SCFAs, are well-documented for their profound impact on host immunity. SCFAs regulate epithelial barrier function and modulate both mucosal and systemic immune responses, primarily via G protein-coupled receptor signalling or histone deacetylase (HDAC) inhibition [13,20]. For instance, SCFAs promote regulatory T-cell differentiation [21], dampen pro-inflammatory cytokine secretion, and enhance immune tolerance, collectively contributing to reduced systemic inflammation [20].

Therefore, this study was designed to systematically evaluate the immunomodulatory potential of *B. breve* MN15965. We first characterized its probiotic properties in vitro, including gastrointestinal tolerance, adhesion capacity, and the immunomodulatory activity of its cell-free supernatant on THP-1 macrophages. Subsequently, we employed a CTX-induced immunosuppression mouse model to investigate the in vivo efficacy of MN15965. The evaluation encompassed its effects on systemic immune indicators (hematological parameters, immune organs, cytokines, and immunoglobulins) and its capacity to modulate the gut environment (fecal SCFA profiles and microbiota composition). Levamisole hydrochloride, a known immunomodulator [22], was used as a positive control to benchmark the strain’s performance.

## 2. Materials and Methods

### 2.1. Bacterial Culture and Preparation

The *B. breve* strain MN15965, originally isolated from the feces of a healthy infant, was provided by Mengniu Dairy. Its taxonomic identity was confirmed via whole-genome sequencing. This specific strain was selected as a favorable candidate for the current study because our extensive preliminary in-house screening of bacterial isolates identified it as a robust producer of indole-3-lactic acid (ILA). Given that ILA is a microbial metabolite well-documented for its potent immunomodulatory properties [23], MN15965 emerged as a highly promising strain for alleviating immune dysfunction. The strain was cultured anaerobically in de Man, Rogosa and Sharpe (MRS) broth (Hope Bio-Technology Co., Ltd., Qingdao, China) supplemented with lithium mupirocin at 37 °C for 24 h to selectively isolate *Bifidobacterium* species from infant feces. After purification, the isolate was routinely cultured in standard MRS broth without any antibiotics for all subsequent experiments. After this standard cultivation, the bacterial culture was centrifuged at 8000× *g* for 5 min at 4 °C, and the antibiotic-free supernatant was filter-sterilized using a 0.22 μm membrane filter.

### 2.2. In Vitro Evaluation of Gastrointestinal Tolerance and Adhesion Capacity

Using a known probiotic *B. breve* M-16V as control, the tolerance of MN15965 to simulated gastrointestinal stresses and its adhesion capacity were assessed. The strain acid tolerance was evaluated by monitoring the bacterial growth in MRS broth at various pH levels of 2, 3, 4, and 5. Each pH-adjusted medium was inoculated with 2% of the MN15965 culture and subsequently incubated anaerobically at 37 °C for 12 h. During incubation, the optical density at 600 nm (OD600) was measured every 30 min to generate growth curves, from which the survival rate under each pH condition was calculated. For bile salt tolerance assessment, an MN15965 culture (2%) was inoculated into MRS broth containing 0%, 0.15%, 0.3%, and 0.6% oxgall. The cultures were incubated under anaerobic conditions at 37 °C for 12 h, and the survival rate was calculated based on OD600 measurements. The strain’s resistance to lysozyme was evaluated in artificial gastrointestinal fluid. The strain was first cultured overnight in MRS broth. Cells were then harvested by centrifugation, washed, and resuspended in the artificial fluid containing lysozyme. Following incubation, the survival rate was measured and compared with that of a control strain to determine its resistance.

The adhesion capacity of *B. breve* MN15965 to mucin was assessed as previously described [24], using the known probiotic strain *B. breve* M-16V [25] as a control. M-16V is a probiotic strain isolated from the fecal sample of a healthy infant [26]. Briefly, porcine gastric mucin-coated wells were incubated for 2 h at 37 °C with MN15965 cultures adjusted to an OD of 1.0 at 620 nm. After incubation, the wells were washed to remove non-adherent bacteria. The remaining adhered bacteria were stained with crystal violet and quantified by measuring the absorbance at 595 nm.

### 2.3. In Vitro Immunomodulatory Activity Assay

The immunomodulatory potential of the MN15965 strain was evaluated using the human monocytic THP-1 cell line, obtained from the Cell Bank of the Chinese Academy of Sciences. THP-1 cells were first differentiated into activated M0 macrophages by treatment with phorbol 12-myristate 13-acetate (100 ng/mL) for 48 h in complete culture medium, and then seeded into 96-well plates at a density of 5 × 10^4^ cells/mL [27]. MRS medium and MN15965 strain culture supernatant were added at 5% as carbon source culture supernatant, while simultaneously inducing polarization to M1 macrophages with LPS (100 ng/mL) and IFN-γ (20 ng/mL). After 24 h, nitric oxide (NO) production was measured using a Nitric Oxide Assay Kit (Beyotime Biotechnology, Shanghai, China) and corrected. Additionally, the concentrations of secreted cytokines (IL-6, TNF-α, IL-10, IL-1β, G-CSF, and GM-CSF) in the supernatant were measured using ELISA kits (YOBIBIO (Shanghai) Biotechnology Co., Ltd., Shanghai, China) according to the manufacturer’s instructions.

### 2.4. Animal Experiment and Sample Collection

All animal procedures were approved by the Animal Ethics Committee of Zhejiang Academy of Agricultural Sciences (Approval No. 25ZALAS07) and conducted in strict accordance with the Guide for the Care and Use of Laboratory Animals. Five-week-old male BALB/c mice, purchased from Jiangsu Jicui Yaokang Biotechnology Co., Ltd., Nanjing, China (License No. SCXK (Su) 2023-0009), were housed under specific pathogen-free (SPF) conditions with a controlled temperature of 22 ± 2 °C, relative humidity of 55 ± 5%, and a 12-h light/dark cycle.

**Figure 1:**
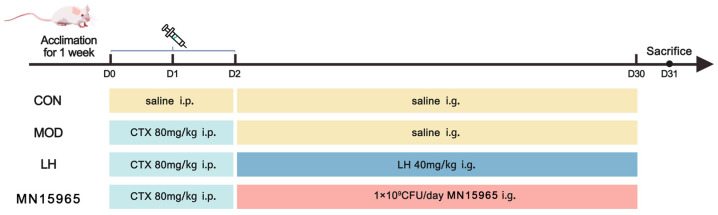
Experimental design (*n* = 6 for each group). CON, normal control group; MOD, CTX-treated model group; LH, levamisole hydrochloride; MN15965, *B. breve* MN15965.

Following a one-week acclimatization period, mice were randomly assigned to 4 groups (*n* = 6), as depicted in Figure 1: normal control (CON) group, CTX-treated model (MOD) group, CTX with levamisole hydrochloride (LH) treatment group, and CTX with MN15965 (MN15965) treatment group. From day 1 to day 3, mice in the MOD, LH, and MN15965 groups received intraperitoneal (i.p.) injections of CTX (80 mg/kg/day), while the CON group received sterile saline (i.p.). Subsequently, from day 4 to day 31, treatments were administered via oral gavage: the CON and MOD groups received sterile saline; the LH group received levamisole hydrochloride (80 mg/kg/day); and the MN15965 group received 1 × 10^9^ CFU of *B. breve* MN15965 per day [28]. To prepare the daily oral dose, fresh bacterial cultures were centrifuged and washed twice with sterile saline to explicitly remove the supernatant. The bacterial pellets were then resuspended in sterile saline to achieve the target dose. During the feeding period, the body weight of the mice was monitored weekly. On day 32, mice were anesthetized with Tribromoethanol. Blood was collected via retro-orbital venous plexus puncture, centrifuged at 1500 rpm for 10 min to obtain serum. Spleens, thymuses, colons, and fecal samples were harvested and stored at −80 °C for subsequent analysis.

### 2.5. Determination of Immune Organ Index

The collected thymus and spleen were subsequently measured. The organ index was calculated using the formula

$$\mathrm{Index}\;\left(\frac{\mathrm{mg}}{g}\right)=\frac{\mathrm{Organ}\;\mathrm{weight}\;\left(\mathrm{mg}\right)}{\mathrm{Final}\;\mathrm{body}\;\mathrm{weight}\;\left(g\right)}$$

### 2.6. Analysis of Hematological and Systemic Immune Parameters

Blood samples were subjected to hematological and immunological analyses. For the hematological analysis, 100 μL of collected blood sample was analyzed using automatic biochemical analyzer (Minday, Shenzhen, China) to determine the counts of white blood cells, lymphocytes, neutrophils, and other key parameters. In parallel, the concentrations of cytokines—including interleukin-6 (IL-6), IL-10, IL-12, tumor necrosis factor-alpha (TNF-α), and interferon-gamma (IFN-γ)—were measured using a Luminex^®^ multiplex assay kit (Wuhan USCN Business Co., Ltd., Wuhan, China). Concurrently, the levels of immunoglobulin A (IgA) and immunoglobulin G (IgG) were determined using commercial enzyme-linked immunosorbent assay (ELISA) kits (Beijing Solarbio Science & Technology Co., Ltd., Beijing, China). All immuno-assays were performed in accordance with the respective manufacturers’ protocols.

### 2.7. Fecal 16S rRNA Gene Sequencing Analysis

For in-depth microbiota analysis, the fecal genomic DNA was extracted using a commercial kit (Tiangen Biotechnology Co., Ltd., Beijing, China). The V3-V4 hypervariable regions of the prokaryotic 16S rRNA gene were amplified using the forward primer 341F (5′-CTACGGGGGCWGCAG-3′) and reverse primer 805R (5′-GATACHVGGGTATCTAATCC-3′). High-throughput sequencing of the amplicons was performed on an Illumina NextSeq platform by LC-Bio Technology Co., Ltd. (Hangzhou, China). Briefly, raw sequencing reads were first processed with fastp [29] for quality filtering and adapter trimming. Subsequently, the DADA2 [30] pipeline was employed to denoise, merge paired-end reads, remove chimeras, and generate a feature table of amplicon sequence variants (ASVs). The ASVs and abundance matrix were imported to QIIME2 [31] for taxonomy assignment and phylogenetic tree construction using the qiime phylogeny align-to-tree-mafft-fasttree pipeline. For alpha and beta diversity comparisons, all samples were rarefied to an even sequencing depth of 40,000 reads. Taxonomic assignment for each ASV was performed using a pre-trained Naïve Bayes classifier against the Greengenes2 database [32].

### 2.8. Short-Chain Fatty Acid Analysis

The concentrations of SCFAs were determined by gas chromatography (GC) according to established methods with modifications [33]. Briefly, for sample preparation, 0.05 g of fecal sample was suspended in Phosphate-Buffered Saline (PBS) at a 1:10 (*w*/*v*) ratio, vortexed thoroughly, and centrifuged at 6000 rpm for 8 min. The supernatant was mixed with a mixture of metaphosphoric acid and crotonic acid, and acidified at −80 °C for 24 h. After acidification, the supernatant was filtered through a 0.22 μm membrane filter prior to GC quantification.

### 2.9. Statistical Analysis

Statistical analysis was performed with one-way analysis of variance (ANOVA) with Tukey’s honestly significant difference test to compare differences between groups using SPSS (version 27.0, IBM Corp., Armonk, NY, USA). Figures were plotted with GraphPad Software Prism 9.5.0 (GraphPad Software Inc., Boston, MA, USA). Data are presented as mean ± SEM. *p* < 0.05 was considered statistically significant. Asterisks denote statistically significant differences (* *p* < 0.05, ** *p* < 0.01, *** *p* < 0.001). Spearman’s correlation coefficient was used to evaluate the relationship between the specific bacteria taxa and levels of SCFAs and cytokines.

## 3. Results

### 3.1. B. breve MN15965 Exhibits Favorable Gastrointestinal Tolerance and Adhesion Capacity In Vitro

**Figure 2:**
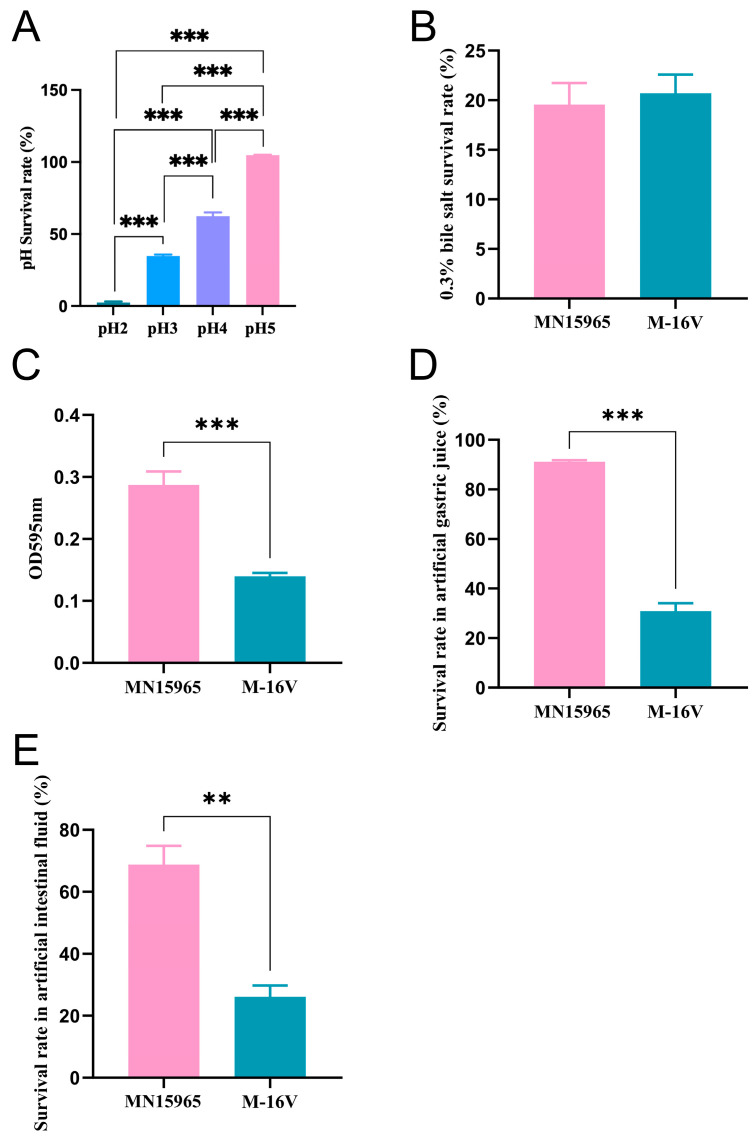
Evaluation of acid, bile salts, and lysozyme tolerance and adhesion capacity of *B. breve* MN15965. (**A**) Survival percentage of *B. breve* MN15965 at different pH concentrations. (**B**) Survival percentage of MN15965 at 0.3% bile salt concentrations. (**C**) Adhesion level to mucin expressed as OD595nm value. (**D**) Survival rate in artificial gastric juice. (**E**) Survival rate in artificial intestinal fluid. MN15965, *B. breve* MN15965; M-16V, *B. breve* M-16V. All data are presented as mean ± SEM. ** *p* < 0.01, *** *p* < 0.001.

To determine if *B. breve* MN15965 is a viable candidate for oral administration before evaluating its in vivo immunomodulatory potential, we first assessed its essential probiotic properties according to established screening guidelines [34]. Therefore, we first evaluated the *B. breve* MN15965’s tolerance to acid condition, bile salt, and gastrointestinal fluids, as well as the ability to colonize the intestinal surface, since these are essential for probiotics to function in the human gastrointestinal tract. As shown in Figure 2A, *B. breve* MN15965 achieved the highest survival rate (>90%) at pH 5 and maintained a survival rate exceeding 50% at pH 4, suggesting considerable acid tolerance. Regarding bile salt tolerance (Figure 2B), the growth dynamics of *B. breve* MN15965 were comparable to that of a known probiotic strain M-16V under the 0.3% bile salt conditions. Notably, its adhesion capacity (*p* < 0.001, Figure 2C) and survival rates in artificial gastric juice (*p* < 0.001, Figure 2D) and artificial intestinal juice (*p* < 0.01, Figure 2E) were significantly higher than those of *B. breve* M-16V. The finding indicates its promising colonization capacity.

### 3.2. Effects of MN15965 on Nitric Oxide Release and Cytokine Secretion Levels in THP-1 Macrophages

**Figure 3:**
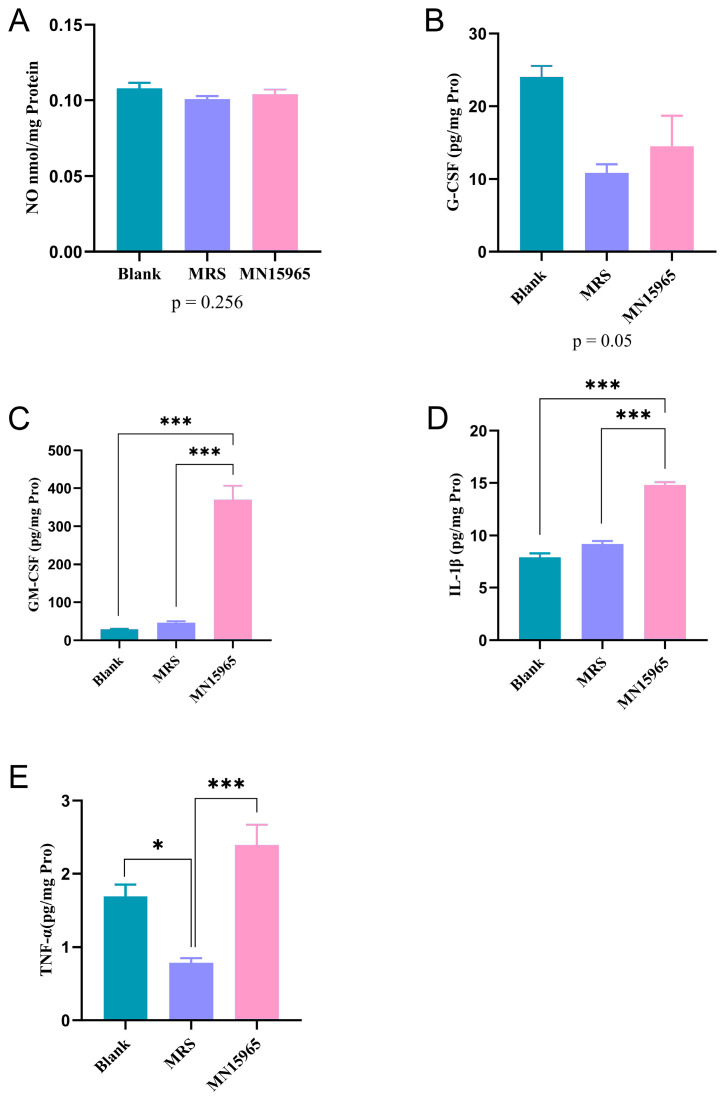
Effects of MN15965 treatment on nitric oxide release and cytokine secretion levels in THP-1 macrophages. (*n* = 6 for each group). (**A**) Nitric oxide (NO). (**B**) G-CSF. (**C**) GM-CSF. (**D**) IL-1β. (**E**) TNF-α. BLANK, blank control group; MRS, MRS medium control group; MN15965, *B. breve* MN15965 treatment group. All data are presented as mean ± SEM. * *p* < 0.05, *** *p* < 0.001.

Building on the strain’s probiotic potential, we next evaluated the effect of its cell-free supernatant on immune cells in vitro (Figure 3). To account for the background effects of the culture medium, we compared the cytokine profiles among the Blank, MRS broth control, and MN15965 groups. As shown in Figure 3A, no significant differences in NO production by macrophages were observed among the Blank, MRS, and MN15965 groups. Compared to the Blank group, the MRS group significantly downregulated the levels of TNF-α (*p* < 0.05, Figure 3E), inhibited the secretion of G-CSF (Figure 3B), and markedly upregulated IL-10 expression (*p* < 0.001), along with an upward trend in the levels of GM-CSF and IL-1β. Therefore, the MRS group served as the baseline to evaluate the true effects of the bacterial metabolites. By contrast, MN15965 intervention enhanced the secretion of GM-CSF (*p* < 0.001, Figure 3C), IL-1β (*p* < 0.01, Figure 3D), and TNF-α (*p* < 0.001, Figure 3E) significantly, and displayed a promoting effect on G-CSF expression.

### 3.3. MN15965 Modulates Host Physiological Parameters and Immune Organ Integrity

**Figure 4:**
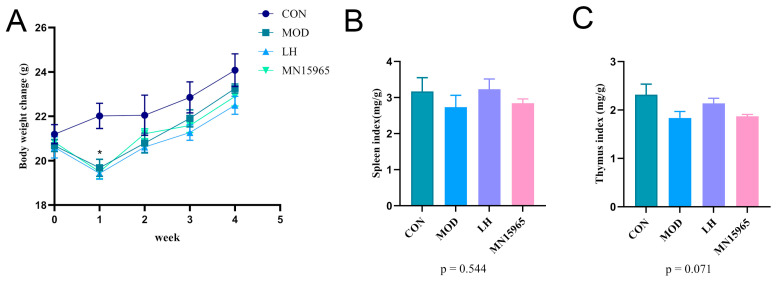
Effects of MN15965 treatment on body weight, and immune organ indices (*n* = 6 for each group). (**A**) Body weight. (**B**) Spleen index. (**C**) Thymus index. CON, normal control group; MOD, CTX-treated model group; LH, levamisole hydrochloride; MN15965, *B. breve* MN15965. All data are presented as mean ± SEM.

Body weight was monitored throughout the study (Figure 4A). Following intraperitoneal injection of CTX, the mice in the MOD, LH, and MN15965 groups experienced a significant decrease in the first week (*p* < 0.05). By the second week, their body weights had recovered and were comparable to the CON group until the end of the study. Notably, the MN15965 group mice had higher body weight than that of the MOD group and the LH group in the second week. Compared with the CON group, the spleen index of the MOD group showed a decreasing trend (Figure 4B). After treatment with LH and MN15965, the spleen index exhibited an upward trend in both groups, although the difference did not reach statistical significance compared to the CON group. A similar trend was observed for the thymus index (Figure 4C).

**Figure 5:**
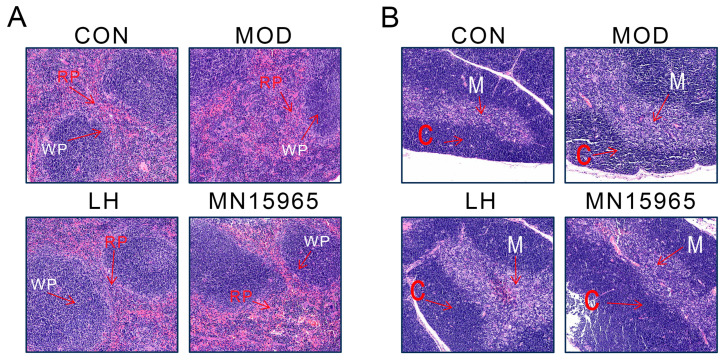
H&E-stained images of spleen and thymus (100 μm). (**A**) spleen section. (**B**) Thymic section. WP: white pulp region of spleen, RP: red pulp region of spleen. M: thymus medulla, C: thymus cortex.

The H&E staining of thymus and spleen confirmed that *B. breve* MN15965 ameliorated CTX-induced organ damage (Figure 5). In the MOD group, the thymus showed a blurred corticomedullary boundary with lymphocyte depletion, and the spleen displayed indistinct red and white pulp regions. Treatment with MN15965 largely reversed these pathological changes, restoring clearer demarcation in the thymus and mitigating the structural disruption in the spleen, bringing their morphology closer to that of the healthy CON group.

### 3.4. B. breve MN15965 Modulates Systemic Hematological and Immune Parameters

The successful establishment of the immunosuppressed model was primarily confirmed by the significant body weight loss in the MOD group during the first week (Figure 4A). The hematological parameters presented here, measured at week 4, likely reflect the early recovery phase post-CTX injection (Table 1). Compared with the CON group, the MOD group showed a downward trend in RBCs, HGB, HCT, MPV, P-LCR, WBCs, and LYM, indicative of CTX-induced myelosuppression. The LH group showed remarkably higher HCT level than MOD group (*p* < 0.05). Additionally, the P-LCR level of MN15965 group (*p* < 0.05) increased significantly in stark contrast to the mice in the MOD group.

Notably, administration of *B. breve* MN15965 showed an upward trend in the levels of both IgA and IgG compared to the MOD group (Table 2). Following LH intervention, the IgA level in mice, was lower than that in the MOD group (*p* < 0.01), while the IgG level remained unchanged. Serum TNF-α levels in the LH and MN15965 groups were higher than those in the MOD group, but the differences were not statistically significant.

### 3.5. B. breve MN15965 Reshapes the Gut Microbiota Composition Disrupted by CTX

**Figure 6:**
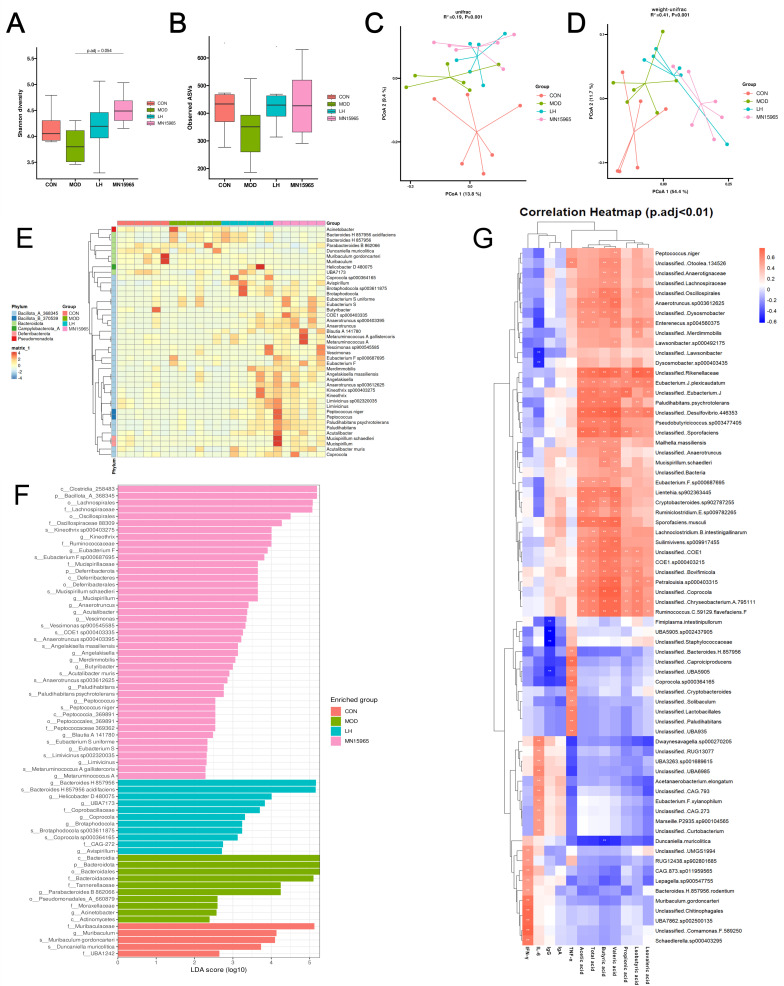
Effects of MN15965 treatment on gut microbiota in CTX-treated mice (*n* = 6 for each group). Alpha diversity estimated by (**A**) Shannon index and (**B**) Observed ASVs. Beta diversity assessed by PCoA based on (**C**) unweighted UniFrac and (**D**) weighted UniFrac distances; statistical significance between groups was assessed using PERMANOVA with BH correction for multiple comparisons. LEfSe analyses of the fecal microbiota in different groups, with differentially abundant taxa (*p*-adjusted < 0.05, LDA score > 2) shown in (**E**) heatmap and (**F**) bar plot at genus and species levels. (**G**) Spearman’s correlation analysis between gut microbiota taxa, short-chain fatty acids (SCFAs), and immune indicators. CON, normal control group; MOD, CTX-treated model group; LH, levamisole hydrochloride; MN15965, *B. breve* MN15965. ** *p* < 0.01.

CTX treatment reduced microbial alpha diversity, as evidenced by a declining trend in both community richness (Observed ASVs) and diversity (Shannon index) in the MOD group compared to the CON group (Figure 6A,B). Both LH and MN15965 interventions reversed this trend, with *B. breve* MN15965 showing a more pronounced improvement in CTX-dysbiosis (*p*-adjusted = 0.054). Analysis of beta diversity by PCoA showed distinct clustering among the groups (Figure 6C,D). Using unweighted UniFrac, the MOD group was separated from both the CON (PERMANOVA, *p*-adjusted = 0.052) and MN15965 groups (*p*-adjusted = 0.052). The weighted UniFrac analysis confirmed this separation with greater statistical significance, revealing pronounced differences between the CON and MOD groups (*p*-adjusted = 0.043) and particularly between the MOD and MN15965 groups (*p*-adjusted = 0.013).

CTX led to a bloom of taxa annotated according to the Greengenes2 database (Figure S1), such as *Bacteroides* H 857956, *Duncanella*, and Unclassified *Muribaculaceae*, and a reduction in potentially beneficial genera like *Ligilactobacillus* and *Alloprevotella*. Compared with the MOD group, LH decreased Unclassified *Muribaculaceae* and increased Unclassified *Lachnospiraceae*. In the MN15965 group, the abundance of *Bacteroides* H 857956, *Parabacteroides* B 862066, *Prevotella* and Unclassified *Muribaculaceae* was reduced, while that of *Alloprevotella*, *Ligilactobacillus* and Unclassified *Lachnospiraceae* was elevated.

LEfSe analysis showed that CTX treatment primarily enriched opportunistic bacteria like *Acinetobacter* and *Duncanella* (Figure 6E,F). In contrast, the MN15965 group was significantly characterized by the enrichment of several beneficial taxa, including the known SCFA producers *Blautia* and *Metaruminococcus*, as well as *Mucispirillum* and members of the *Eubacterium* family.

### 3.6. B. breve MN15965 Selectively Increased Fecal Levels of Short-Chain Fatty Acids

**Figure 7:**
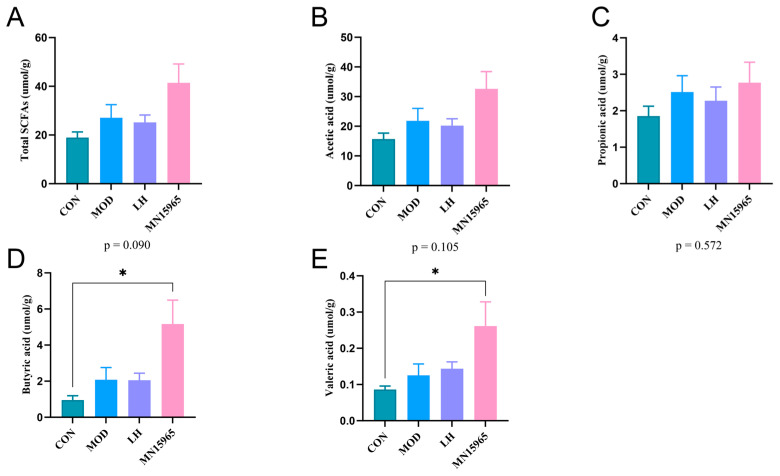
Effects of MN15965 treatment on SCFAs in CTX-treated mice (*n* = 6 for each group). (**A**) Total SCFAs. (**B**) Acetic acid. (**C**) Propionic acid. (**D**) Butyric acid. (**E**) Valeric acid. CON, normal control group; MOD, CTX-treated model group; LH, levamisole hydrochloride; MN15965, *B. breve* MN15965. All data are presented as mean ± SEM. * *p* < 0.05.

The MN15965 group tended toward increased SCFA levels compared with the MOD group, yet no statistically significant differences were observed (*p* = 0.09). Remarkably, in contrast to the CON group, the levels of butyric acid (*p* < 0.05) and valeric acid (*p* < 0.05) were significantly increased in mice receiving MN15965 treatment (Figure 7).

### 3.7. Correlation Analysis Between Gut Microbiota, SCFAs, and Immune Indicators in Mice

Spearman’s correlation analysis revealed significant associations between microbial taxa and host parameters (Figure 6G). Taxa enriched in the MOD group, such as *Bacteroidaceae* and *Parabacteroides* B 862066, were negatively correlated with valeric acid (*p*-adjusted < 0.01) and positively correlated with pro-inflammatory cytokines IL-6 and IL-10 (*p*-adjusted < 0.01). Conversely, key bacteria enriched by the MN15965 treatment such as *Eubacterium* F sp000687695, *Mucispirillum schaedleri* and *Anaerotruncus* sp000403395, were positively correlated (*p*-adjusted < 0.01) with acetic acid, isobutyric acid, butyric acid, valeric acid, and total acid.

## 4. Discussion

This study aimed to evaluate the immunomodulatory potential of a novel strain, *B. breve* MN15965. Our in vitro tolerance assays confirmed that *B. breve* MN15965 possesses the fundamental characteristics required for a probiotic. It demonstrated favorable tolerance to acid and bile salts, along with a marked capacity for adhesion to intestinal epithelial cells. Notably, its survival rate of over 60% in simulated gastrointestinal fluid was higher than the commercial probiotic strain, *B. breve* M-16V [26]. These results, consistent with the previous findings [35], indicate that MN15965 has substantial potential to survive gastrointestinal transit and colonize the intestine, which is a prerequisite for exerting its biological functions.

To characterize its immunomodulatory properties, the effects of the MN15965 supernatant on immune cells were evaluated using in vitro cell culture assays. MN15965 not only reversed the inhibition of TNF-α in the MRS broth but also significantly upregulated the secretion of GM-CSF and IL-1β. This cytokine profile is highly significant. The upregulation of TNF-α and IL-1β indicates a robust activation of THP-1 macrophages towards a pro-inflammatory M1 phenotype [36]. In the context of an immunosuppressed host, this M1 polarization is not detrimental; rather, it represents a crucial reactivation of the innate immune system, stimulating a defensive response capable of clearing pathogens or damaged cells [37]. This activation of macrophage inflammatory pathways is a well-documented immunomodulatory mechanism for many probiotic strains, including various *Lactobacillus* and *Bifidobacterium* species, which often engage innate immune receptors like TLRs [38,39,40].

Based on the in vitro evidence of immunomodulatory potential, the strain’s systemic immunomodulatory effects were assessed in a CTX-induced immunosuppression mouse model. CTX, a broad-spectrum alkylating agent, is known to induce immune dysfunction by inhibiting immune cell proliferation [7] and damaging the intestinal barrier [41]. In our model, CTX successfully induced rapid weight loss in the first week, reduced immune organ (spleen and thymus) indices, and caused abnormal hematological parameters, phenotypes consistent with previous reports [42]. Interestingly, CTX did not cause a consistent decrease in serum immunoglobulins and cytokine levels by the end of the study (day 32). This discrepancy likely underscores the importance of temporal dynamics in this model. While CTX successfully induced acute injury, the prolonged intervention period (day 4 to day 31) may have allowed for partial spontaneous immune recovery in the MOD group. This is consistent with previous findings that CTX-induced lymphopenia is often transient [43]. Additionally, the dual effects of CTX at different concentrations [44] might also be a contributing factor: while inducing immunosuppression, it also causes depletion of Tregs, which can paradoxically activate effector T cells [45]. The cytokines produced by these activated effector T cells might have partially offset the immunosuppression, leading to no significant changes observed at the serum level.

The restorative effects of MN15965 on systemic immunity were evident at multiple levels. After treatment with LH and MN15965, body weight, spleen index, and thymus index in mice were restored toward normal levels. H&E staining visually confirmed the protective effects of MN15965 on the histological structures of the thymus and spleen, consistent with findings on heat-inactivated BBMN68 by Zhou et al. [46]. Furthermore, MN15965 mitigated CTX-induced myelosuppression, as indicated by the recovery trend in P-LCR levels, consistent with observations that the gut microbiota can influence bone marrow hematopoiesis through the systemic release of bacterial metabolites [47,48]. This suggests a potential “gut–bone marrow axis” mechanism mediated by MN15965. Regarding immunoglobulins, levamisole hydrochloride was selected strictly based on its historical widespread use in animal models for evaluating immunostimulatory agents [49,50]. As an anthelmintic agent with known risks of agranulocytosis, its inherent toxicity corroborates our hematological data. The LH group exhibited a significant reduction in serum IgA levels compared to the MOD group. In contrast, MN15965 intervention restored both serum IgA and IgG levels to near those of the CON group. This suggests that MN15965 may promote B-cell differentiation and IgA secretion by activating antigen-presenting cells (such as dendritic cells) in Peyer’s patches [51], while potentially boosting IgG levels by modulating the Th1/Th2 balance via pathways like JAK-STAT [52].

Recently, numerous studies have established that the immunomodulatory functions of probiotics are closely linked to their regulation of the gut microbiota [18,53]. In our study, MN15965 intervention significantly reshaped the gut microecology disrupted by CTX. Compared to the MOD group, the Shannon index in the MN15965 group showed a marginally significant improvement (*p*-adjusted = 0.054), and β-diversity analysis revealed that its community structure shifted away from the MOD group and towards the CON group. This structural modulation is frequently associated with attenuated intestinal inflammation and the restoration of host homeostasis [54]. LEfSe analysis further identified that MN15965 enriched several beneficial taxa such as *Lachnospiraceae, Eubacterium* [55], while suppressing opportunistic pathogens like *Acinetobacter* [56]. This likely reflects the restoration of intestinal hypoxia, which inhibits the bloom of facultative anaerobes driven by inflammation [57].

Crucially, the immunomodulatory effects of MN15965 appear to be mediated by specific microbial metabolites. Although the total SCFA levels did not differ significantly between the MN15965 and MOD groups, the levels of butyric acid and valeric acid were notably higher in the MN15965 group than in the CON group (*p* < 0.05). Butyric acid is a primary energy source for colonocytes and a key regulator of intestinal barrier function, inflammation and Treg differentiation [21]. Specifically, intestinal mucosal integrity, which is typically evaluated via trans epithelial electrical resistance, is highly dependent on butyrate to upregulate tight junction proteins such as Claudin-1 and ZO-1 [58,59]. Thus, the pronounced elevation of these SCFAs provides robust functional evidence for intestinal barrier restoration. Valeric acid has also been reported not only to exert anti-inflammatory via HDAC inhibition [60], but also to enhance the effector function of CD8+ T cells and cellular metabolism [61]. The selective accumulation of these SCFAs implies that *B. breve* MN15965 specifically promoted the colonization or activity of a functional guild of SCFA-producing microbiota. We speculate that MN15965 may act to modulate the gut environment by competing for ecological niches or modulating host immunity, potentially driving the reorganization of the intestinal microbiota towards more beneficial functional communities [62]. This is supported by our Spearman’s correlation analysis. The fecal level of butyric acid and other SCFAs were positively correlated with bacterial taxa enriched in the MN15965 group, such as *Eubacterium* F sp000687695, *Mucispirillum schaedleri*, and *Anaerotruncus* sp000403395, suggesting their potential immune-modulatory benefits [63,64]. Notably, our 16S rRNA sequencing did not detect an increase in *Bifidobacterium* abundance in the MN15965 group. This is primarily because exogenous probiotics typically exhibit transient colonization rather than permanent engraftment [65].

Nevertheless, this study has some limitations. Firstly, without an additional bacterial control in vivo, we cannot assess the immunomodulatory effects of *B. breve* MN15965 relative to other strains. Secondly, while the murine model effectively mimics immunosuppression, findings cannot be directly extrapolated to complex human immune disorders [66]. Thirdly, although our study reveals strong correlations between the microbiota, SCFAs, and immune parameters, correlation does not imply causation. Future studies using germ-free mouse transplantation and direct metabolite interventions are needed to establish a causal relationship.

## 5. Conclusions

In conclusion, this study demonstrated that *B. breve* MN15965 is a promising probiotic strain with potent immunomodulatory effects. In a CTX-induced immunocompromised mouse model, MN15965 effectively restored immune homeostasis, evidenced by improvements in physiological indices, hematological parameters, and immunoglobulin levels. Moreover, these benefits were strongly associated with the capacity of MN15965 to enhance gut bacterial diversity, and enrich potentially beneficial SCFA-producing taxa. The present findings underscore the therapeutic potential of *B. breve* MN15965 for individuals with compromised immune function.

## Figures and Tables

**Table 1:** Effects of *B. breve* MN15965 treatment on blood indicators in CTX-treated mice.

Blood Indicators	CON	MOD	LH	MN15965
RBCs (10^12^/L)	7.71 ± 0.26	6.83 ± 0.83	8.43 ± 0.14	8.35 ± 0.16
HGB (g/L)	132.44 ± 5.54	117.60 ± 15.31	149.17 ± 2.56	150.00 ± 3.01
HCT (%)	30.11 ± 1.04 ^ab^	26.70 ± 3.36 ^b^	33.85 ± 0.68 ^a^	32.93 ± 0.65 ^ab^
MCH (pg)	17.14 ± 0.22 ^b^	17.12 ± 0.31 ^ab^	17.70 ± 0.15 ^ab^	17.97 ± 0.20 ^a^
MPV (fL)	11.89 ± 0.22	11.46 ± 0.56	11.58 ± 0.13	12.74 ± 0.32
P-LCR (%)	0.34 ± 0.01 ^ab^	0.33 ± 0.03 ^b^	0.33 ± 0.01 ^b^	0.41 ± 0.02 ^a^
WBCs (10^9^/L)	7.09 ± 1.40	6.28 ± 1.93	4.92 ± 0.96	7.82 ± 1.23
LYM (10^9^/L)	3.90 ± 0.61	2.60 ± 0.50	3.98 ± 0.60	4.16 ± 0.65

RBCs, red blood cells; HGB, hemoglobin; HCT, hematocrit; MCH, mean corpuscular hemoglobin; MPV, mean platelet volume; P-LCR, platelet large cell ratio; WBCs, white blood cells; LYM, lymphocyte. Data are expressed as mean ± SEM (*n* = 6). Different lowercase superscript letters (a, b) within the same row indicate significant differences (*p* < 0.05) among groups. The absence of superscript letters indicates no significant differences between groups.

**Table 2:** Effects of *B. breve* MN15965 treatment on immunoglobulin levels and serum cytokines in CTX-treated mice.

Indicators	CON	MOD	LH	MN15965
IgA (pg/mL)	194.13 ± 25.30 ^a^	219.53 ± 33.19 ^a^	89.73 ± 10.43 ^b^	273.25 ± 32.43 ^a^
IgG (μg/mL)	2.10 ± 0.02 ^a^	1.99 ± 0.04 ^ab^	1.93 ± 0.06 ^b^	2.12 ± 0.01 ^a^
TNF-α (pg/mL)	16.19 ± 0.50 ^b^	17.13 ± 0.29 ^ab^	20.73 ± 1.22 ^a^	17.59 ± 1.20 ^ab^

IgA, Immunoglobulin A; IgG, Immunoglobulin G; TNF-α, Tumor Necrosis Factor alpha. Data are expressed as mean ± SEM (*n* = 6). Different lowercase superscript letters (a, b) within the same row indicate significant differences (*p* < 0.05) among groups using ANOVA.

## Data Availability

The data presented in this study are openly available in NCBI Sequence Read Archive database at https://www.ncbi.nlm.nih.gov/bioproject/PRJNA1387360 (accessed on 19 January 2026), reference number PRJNA1387360.

## Supplementary Materials

The following supporting information can be downloaded at: https://www.mdpi.com/article/10.3390/microorganisms14050949/s1, Figure S1: The effects of MN15965 on the structure and composition of the gut microbiota.
