# Supplementary material for: Bifidobacterium breve MN15965 Improved Bacterial Diversity, Short-Chain Fatty Acid Production, and Immune Activation in a Cyclophosphamide-Induced Immunosuppression Mouse Model
Source: Microorganisms. 2026 Apr 23;14(5):949. doi: 10.3390/microorganisms14050949 (PMC13210003; doi:10.3390/microorganisms14050949)
Supplement: Supplementary file 1 [file microorganisms-14-00949-s001.zip › microorganisms-4134453-supplementary.pdf]

Supplementary materials for

Bifidobacterium breve MN15965 improved bacterial diversity, short-chain fatty acid production, and immune activation in a cyclophosphamide-induced immunosuppression mouse model

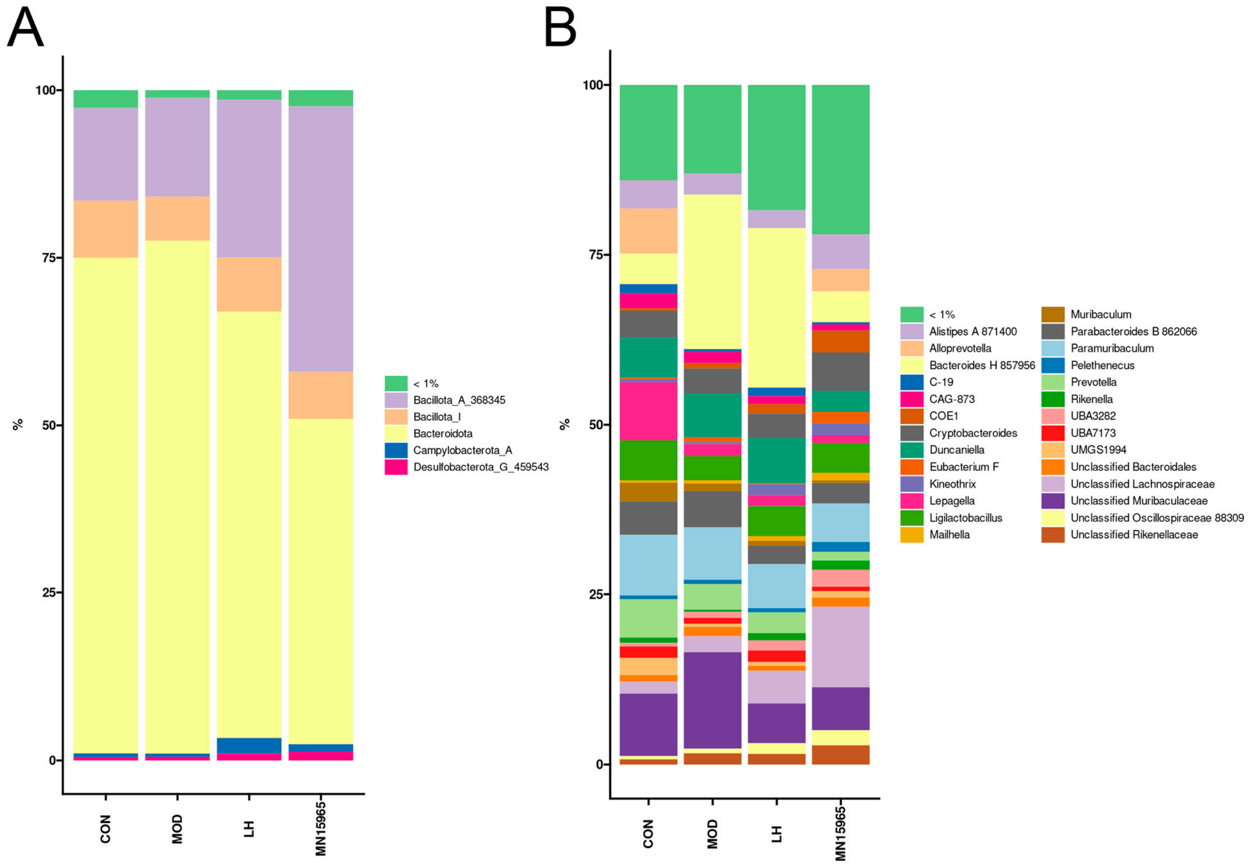

Supplementary Figure S1. The effects of MN15965 on the structure and composition of the gut microbiota. (*n* = 6 for each group). **(A)** Phylum level. **(B)** Genus level. CON, normal control group; MOD, CTX-treated model group; LH, levamisole hydrochloride; MN15965, *B, breve* MN15965.
